# Treatment of ADHD: Drugs, psychological therapies, devices, complementary and alternative methods as well as the trends in clinical trials

**DOI:** 10.3389/fphar.2022.1066988

**Published:** 2022-11-17

**Authors:** Victoria A. Nazarova, Aleksandr V. Sokolov, Vladimir N. Chubarev, Vadim V. Tarasov, Helgi B. Schiöth

**Affiliations:** ^1^ Department of Surgical Sciences, Functional Pharmacology and Neuroscience, Uppsala University, Uppsala, Sweden; ^2^ Advanced Molecular Technology LLC, Moscow, Russia

**Keywords:** ADHD, treatment, pharmacological, non-pharmacological, trials, stimulants, agents, drugs

## Abstract

Attention-deficit/hyperactivity disorder (ADHD) is one of the most common neurodevelopmental disorders having a high influence on social interactions. The number of approved treatments and clinical trials for ADHD have increased markedly during the recent decade. This analytical review provides a quantitative overview of the existing pharmacological and non-pharmacological methods of ADHD treatments investigated in clinical trials during 1999–2021. A total of 695 interventional trials were manually assessed from clinicaltrial.gov with the search term « ADHD», and trial data has been used for analysis. A clear majority of the studies investigated non-pharmacological therapies (∼80%), including many behavioral options, such as social skills training, sleep and physical activity interventions, meditation and hypnotherapy. Devices, complementary and other alternative methods of ADHD treatment are also gaining attention. The pharmacological group accounts for ∼20% of all the studies. The most common drug classes include central nervous system stimulants (e.g., methylphenidate hydrochloride, lisdexamfetamine dimesylate, amphetamine sulfate, mixed amphetamine salts, a combination of dexmethylphenidate hydrochloride and serdexmethylphenidate chloride), selective noradrenaline reuptake inhibitors (atomoxetine, viloxazine), and alpha2 adrenergic receptor agonists (guanfacine hydrochloride, clonidine hydrochloride). Several studies investigated antidepressants (e.g., bupropion hydrochloride, vortioxetine), and atypical antipsychotics (e.g., quetiapine, aripiprazole) but these are yet not approved by the FDA for ADHD treatment. We discuss the quantitative trends in clinical trials and provide an overview of the new drug agents and non-pharmacological therapies, drug targets, and novel treatment options.

## Introduction

Attention-deficit/hyperactivity disorder (ADHD) is a neurobehavioral disorder, which can be reliably diagnosed in children, adolescents, and adults (Wilens and Spencer, 2010; Brown et al., 2018). The disorder affects around 5% of children and adolescents (Song et al., 2021) and 2.5% of adults (Simon et al., 2009) worldwide. There are three main ADHD subtypes, which are based on the prevalence of inattention or hyperactivity-impulsivity symptoms according to the Diagnostic Statistical Manual of fifth revision (DSM-5): predominantly inattentive type (ADHD-I), predominantly hyperactive-impulsive type (ADHD-H), combined type (ADHD-C) (Ayano et al., 2020; Diagnostic and statistical manual of mental disorders, 2013). Individuals with ADHD usually tend to experience anxiety and chronic stress, have poor relationships with peers and parents and also have employment problems. Moreover, they have lower education attainment, are easily distracted, are prone to substance misuse, and more frequently get in serious road traffic accidents (Alternative treatments for attention deficit hyperactivity disorder, 2003; Caye et al., 2019).

ADHD has been associated with genetic, neurological and environmental factors and combinations thereof (Akutagava-Martins et al., 2016), but the exact pathophysiology of ADHD is still not clear (Thapar and Cooper, 2016). Most often the disease has been associated with abnormalities in neurotransmitter regulations, such as dopamine and norepinephrine. Many of these neurotransmitters mediate their effects through G-protein coupled receptors (GPCRs) (Lagerstrom and Schioth, 2008; Hauser et al., 2017) and are transported by corresponding transporters (Fredriksson et al., 2008; Rask-Andersen et al., 2013); both of which are important classes of drug targets. Moreover, specific brain structures such as the prefrontal cortex (PFC), caudate, basal ganglia, anterior cingulate cortex, and cerebellum have been implicated in the disease pathology. Functional MRI studies have shown differences in the structural development and functional activation of these structures in patients with ADHD (Poelmans et al., 2011; Sharma and Couture, 2014). Many of these brain areas and their connections are considered to regulate cognitive functions, such as attention, thinking, learning, inhibitory control, impact emotions, behavior, actions, and are associated with many symptoms such as distractibility, forgetfulness, impulsivity, poor planning, and hyperactivity, which are commonly encountered in patients with ADHD (Arnsten, 2006; Arnsten and Pliszka, 2011; Kesner and Churchwell, 2011).

There is a growing number of ADHD treatments that target and alleviate the core symptoms of ADHD and functional impairment (Caye et al., 2019). There are specific guidelines for the stepwise management of ADHD in the UK, Europe, and the US (Thapar and Cooper, 2016). Methods of ADHD treatment can be divided into two main groups: pharmacological and non-pharmacological. The first group includes medications such as stimulants, which are considered to be the first-line pharmacological treatment for ADHD, non-stimulants (the second-line medications), and other approved or unapproved drugs (Catala-Lopez et al., 2015). The last two groups can be used when the first in line stimulants are not well-tolerated or effective. In addition to pharmacological methods, there are non-pharmacological ones that have been extensively investigated over the years (Thapar and Cooper, 2016). This group comprises three main domains: psychological methods, complementary and alternative medicine, and devices.

There are several reviews about the field of ADHD treatment (Sonuga-Barke et al., 2013; Catala-Lopez et al., 2017; Cortese et al., 2018; Lambez et al., 2020; Nimmo-Smith et al., 2020). A systematic review of the pharmacological and non-pharmacological treatment of ADHD in children and adolescents by Catala-Lopez et al. offers a comprehensive and detailed review of 190 studies. It was concluded that behavioral therapy was the only non-pharmacological intervention that had a statistically significant effect in the management of ADHD. Stimulants such as methylphenidate and amphetamine were found to be the most effective amongst the pharmacological interventions for the disease, whereas non-stimulants such as atomoxetine, guanfacine, and clonidine were considered secondary treatment options. Moreover, it was suggested that treatment effects of behavioral therapy were larger when combined with stimulants (Catala-Lopez et al., 2017). In 2018, Cortese et al. analyzed the efficacy and tolerability of medications for ADHD in children, adolescents, and adults. By systematically analyzing data from 133 studies, it was found that methylphenidate in children and adolescents, and amphetamines in adults were the first pharmacological choice for short-term management of ADHD. Apart from the fact that, amphetamines were the most efficacious compounds in adult ADHD patients, such factor as acceptability played a decisive role, as amphetamines were the only compound with better acceptability than placebo in adults and methylphenidate had better acceptability than placebo in children and adolescents (Cortese et al., 2018). This data also perfectly correlates with information from NICE guidelines, which ranks methylphenidate over amphetamines in children and adolescents (National Institute for Health and Care Excellence, 2018). However, the FDA recommend these compounds for ADHD treatment without any distinction.

Comprehensive information about the non-pharmacological methods was presented in a review analyzing 54 studies (Sonuga-Barke et al., 2013). This work includes dietary interventions (restricted elimination diets, artificial food color exclusions, free fatty acid supplementation) and psychological interventions (cognitive training, neurofeedback, behavioral interventions). It was concluded that artificial food color exclusions and free fatty acid supplementation produced a reduction in ADHD symptoms. For other types of therapies evidence of efficacy from blinded assessments was needed (Sonuga-Barke et al., 2013). Moreover, in 2019, Nimmo-Smith et al. conducted a systematic analysis of 32 studies on non-pharmacological interventions. The prevailing number of trials examined cognitive behavior therapy, which showed significant results in a reduction of the core behavioral ADHD symptoms. There were also other interventions, such as cognitive remediation and rehabilitation, mindfulness-based therapies, dialectical behavior therapy, and hypnotherapy that demonstrated some promising results as ADHD treatment (Nimmo-Smith et al., 2020). A systematic review and meta-analysis by Lambez et al. provided valuable information about non-pharmacological methods aimed at cognitive symptomatology in ADHD patients. Physical exercise interventions, especially aerobic ones, were found to be the most effective in targeting and reducing cognitive symptoms of ADHD. Such therapies as cognitive behavioral therapy (CBT), neurofeedback, biofeedback, and cognitive training also appeared to be very effective. Moreover, it was concluded that the most affected cognitive functions were inhibition and flexibility. Taking into consideration studies that combined pharmacological and non-pharmacological therapies, it was noted that there was only slightly increased improvement when medication was used along with a non-pharmacological intervention. Furthermore, studies that included only non-medicated participants still showed promising results (Lambez et al., 2020).

In this review, we provide a quantitative analysis of ADHD treatment methods that have reached clinical trials starting from 1999 to the end of 2020. We aimed to show the diversity of treatment options, their features and the trends looking closer into therapies that seem to be gaining attention.

## Data collection and analysis

All trials included in this review were taken from clinicaltrials.gov–one of the largest web-based resources providing access to summary information on clinical studies on a wide variety of diseases and conditions (Tse et al., 2018). An advanced search was used and only interventional studies were considered. The term “ADHD” was used in the search field “Condition or disease”. Other fields “Country” and “Other terms” were not applied. The following synonyms have been automatically added to the «ADHD » search term by the clinicaltrials.gov search engine: “Attention deficit”, “Hyperactivity disorder”, “Disorder hyperactivity”, “Hyperkinetic disorder”, “Hyperkinetic Syndrome”, “Minimal brain dysfunction”. All ADHD subtypes (e.g., combined type, predominantly inattentive, and predominantly hyperactive-impulsive) were included in the analysis. There were no criteria for inclusion regarding the gender and age of participants. All studies up until 4 January 2021 were analyzed.

The initial search yielded 1,093 trials. The key criterion for including the study in the review was that the study description contained information about the treatment of ADHD by one of the methods: pharmacological, non-pharmacological, or their combinations. The primary purpose of the study should have been treatment. Studies including patients with comorbid conditions (such as anxiety, depression, epilepsy, or other medical conditions) were also eligible for inclusion provided that these conditions were associated with ADHD. Studies with no participants were excluded from the analysis. Thus, by manual selection, 398 studies were excluded and a total of 695 studies were included in this review. Information regarding NCT number, study title, study status, researched condition, intervention, phase of the study, completion date of the study, and date when its results were first posted was collected for each included trial through clincialtrials.gov. Some trials had incorrectly/ambiguously assigned phases, thus requiring their correction. If a study had a double phase (e.g., Phase 1|Phase 2), we took an earlier one since it is not clear whether a trial has successfully transitioned to the next phase. If the phase was categorized as « Not applicable», «Early Phase 1» we classified it as Phase 1. For devices, according to the FDAs classification, the term « Phase» is not correct, thus we used the term « Stage» (e.g., exploratory stage and pivotal stage) (FDA, 2013). Exploratory stage contains pilot, feasibility and first-in-human studies. Study assignment to the stage was based on the number of participants taking part in the clinical study.

We have analyzed and classified treatment methods from the included studies, using classification from previously published articles on the topic (Bader and Adesman, 2012; Esparham et al., 2014; Catala-Lopez et al., 2015; Sharma et al., 2015; Catala-Lopez et al., 2017; Brown et al., 2018; Shrestha et al., 2020).

The following classification was applied:

1) **Pharmacological interventions**:
  - ***stimulant drugs:***
    • Norepinephrine and dopamine reuptake inhibitors (methylphenidate hydrochloride, lisdexamfetamine dimesylate, dexmethylphendate hydrochloride, *etc.*)
  - ***non-stimulant drugs***:
    • Apha2 adrenergic receptor agonists (guanfacine, clonidine)
    • Selective noradrenaline reuptake inhibitor (atomoxetine)
  - ***other approved or unapproved drugs used in ADHD***:
    • Antidepressants (bupropion hydrochloride, venlafaxine hydrochloride, desipramine hydrochloride, *etc.*)
    • Antipsychotics (risperidone, aripiprazole, olanzapine, *etc.*) (Catala-Lopez et al., 2015; Catala-Lopez et al., 2017; Brown et al., 2018)
2) Non-pharmacological interventions:
  - ***psychological:***
    • Behavior management interventions (parent training, classroom interventions, peer-based interventions, classical contingency management, cognitive behavior therapy, organizational skills intervention, social skills training, *etc.*)
    • Cognitive training interventions (cognitive training, working memory training, attention training, neurofeedback, *etc.*)
    • Psychoeducation (Catala-Lopez et al., 2015; Catala-Lopez et al., 2017; Shrestha et al., 2020)
  - ***complementary and alternative methods*** *:*
    • Supplementary interventions and dietary interventions (polyunsaturated fatty acids, vitamins, minerals, amino acids, herbal treatment, homeopathic treatment, *etc.*)
    • Mind-body interventions (meditation, Yoga, Tai chi, mindfulness, hypnotherapy, massage, acupuncture, chiropractic, and osteopathic manipulation, physical activity, *etc.*) (Bader and Adesman, 2012; Esparham et al., 2014; Catala-Lopez et al., 2015; Sharma et al., 2015; Catala-Lopez et al., 2017; Shrestha et al., 2020)
  - ***devices***
    • Transcranial direct current stimulation (tDCS)
    • Transcranial random noises stimulation (tRNS)
    • Repetitive transcranial magnetic stimulation (rTMS)
    • Direct transcranial magnetic stimulation (dTMS)
    • External trigeminal nerve stimulation (eTNS)
    • Virtual reality (VR)
3) Combined interventions

In trials with drugs as an intervention, all information regarding INN, agent type, agent class, target type, mechanism of action, and FDA approval was collected from go.drugbank.com, genome. jp, uniprot.org, and fda.gov in a similar way as we have done previously (Attwood et al., 2020; Attwood et al., 2021).

We did not consider different doses and drug forms, except for transdermal systems (e.g., methylphenidate transdermal system, amphetamine transdermal system). All devices encountered in clinical trials were divided into the corresponding groups presented in the classification. If a device could not be assigned to any of the aforementioned classes, we classified them as « Unspecified device class». If a study contained multiple therapies (e.g., parent training, vitamin, and massage) or a combination of them, each therapy was considered separately in the analysis. It is also important to note, that trends in clinical trials were in our main focus, and the data obtained during the analysis do not correlate with the actual application of methods in clinical practice.

It should be mentioned that these quantitative analyses have some limitations. It was not always clear whether the patient was treated with another ADHD treatment (e.g., drugs, psychological therapies, supplementary interventions) during or prior to the intervention. Thus, some of the interventions that were listed as single might have been combined with other forms of ADHD treatment. Additionally, some trials included several conditions, and therefore it is possible that ADHD was not necessarily the major focus of each trial, which could cause misleading data regarding certain trends. Some drugs were indicated in the trials without specifying their names, hence they could not be attributed to any particular class. In some trials on non-pharmacological methods, on the contrary, there was a name of the therapy class (e.g., behavior parent training, working memory training, tDCS), but based on the description of the study, it was not always clear what particular device (e.g., Soterix Medical tDCS device, Magstim Super Rapid2 stimulator) or therapy (e.g., Parents InC, Cogmed Working Memory Training) were used. In such cases, each therapy and device were considered as a unique entity (e.g., Device_tDCS_1, Device_tDCS_2, Device_rTMS_4, Cognitive training_3, *etc.*). It should be mentioned that the prevailing majority of psychological and complementary and alternative methods belongs to the phase categorized as « Not Applicable». For the sake of simplicity, we classified these studies as « Phase 1», however, this could lead to data misinterpretation. More specific analyses focusing on behavioral therapies should consider detailed classification of these studies. For example, NIH proposes to classify behavioral therapy trials based on the setting of a trial, starting from basic science in the research setting and finishing with trials in community settings (Onken et al., 2014). All clinical trials included in the analysis were manually collected from clinicaltrials.gov database and no other sources and registers of clinical trials were taken into consideration.

## The most commonly used drug classes in clinical trials

**FIGURE 1:**
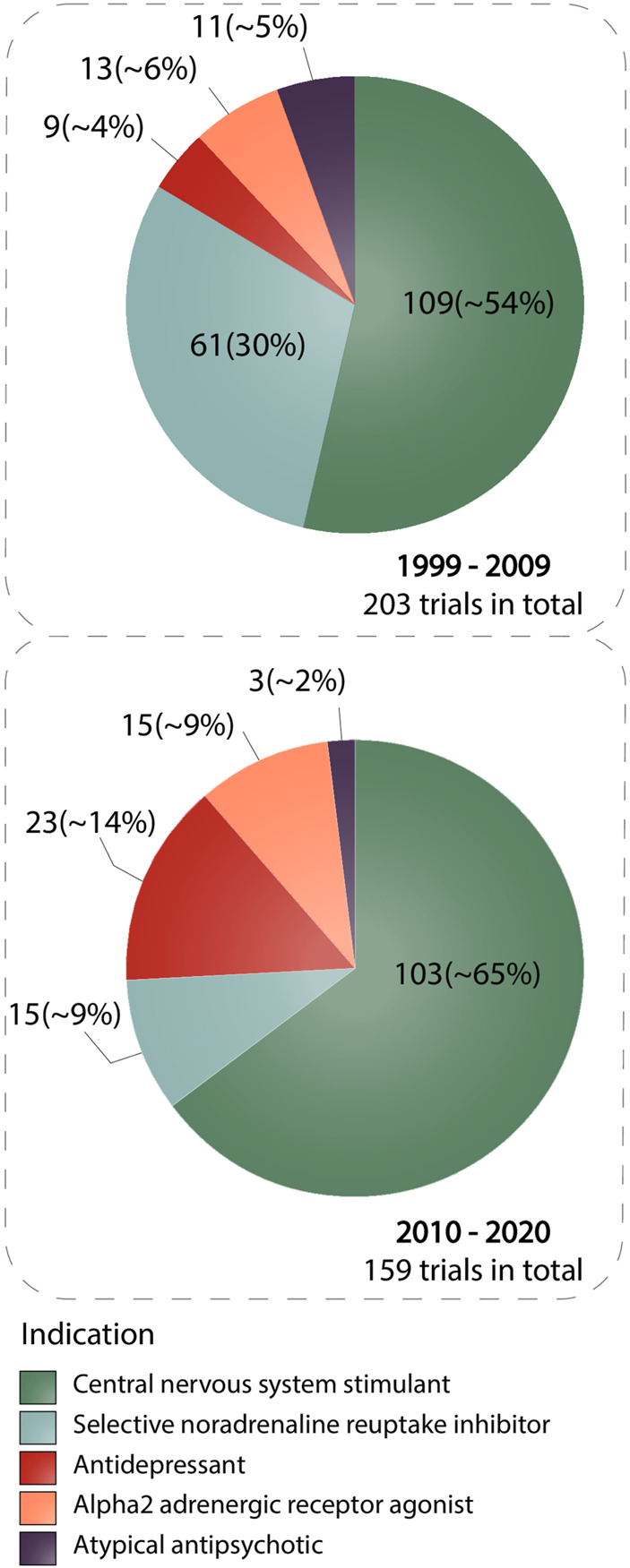
The figure presents information about the number of trials among the most widely used pharmacological classes of drugs applied for ADHD treatment in two time intervals (1999–2009 and 2010–2020). The colors of the slices comply with the pharmacological classes. The data is due 4 January 2021.

Since drugs are considered to be one of the most important parts of ADHD treatment, we illustrated the most commonly used classes of drugs in clinical trials and how their intervention frequency changed over the two periods of time (1999–2009, 2010–2020) (Figure 1). The five most widely used classes included central nervous system stimulants (stimulants), selective noradrenaline reuptake inhibitors, alpha2 adrenergic receptor agonists, antidepressants, and atypical antipsychotics. We noticed a substantial difference in numbers between the stimulants group and among other groups. Central nervous system stimulants appeared in 212 interventions, whereas there were 74 interventions in total for alpha2 adrenergic receptor agonists, antidepressants, and atypical antipsychotics. The second largest class is selective noradrenaline reuptake inhibitors with 76 interventions in both time intervals. For this group, we can see a steep decrease in the number of interventions between 1999–2019 and 2010–2020 time periods, which amounted to 61 and 15 interventions, respectively. A slight drop in the number of studies was also observed in the atypical antipsychotics class, from 11 trials during the 1999 to 2019 timeline to three trials in 2010–2020. In contrast, stimulants and alpha2 adrenergic receptor agonists showed no marked sign of going up or down and remained stable. Only antidepressants demonstrated an increase from nine to 23 interventions in the first and the second time intervals, respectively.

## Drug targets

**FIGURE 2:**
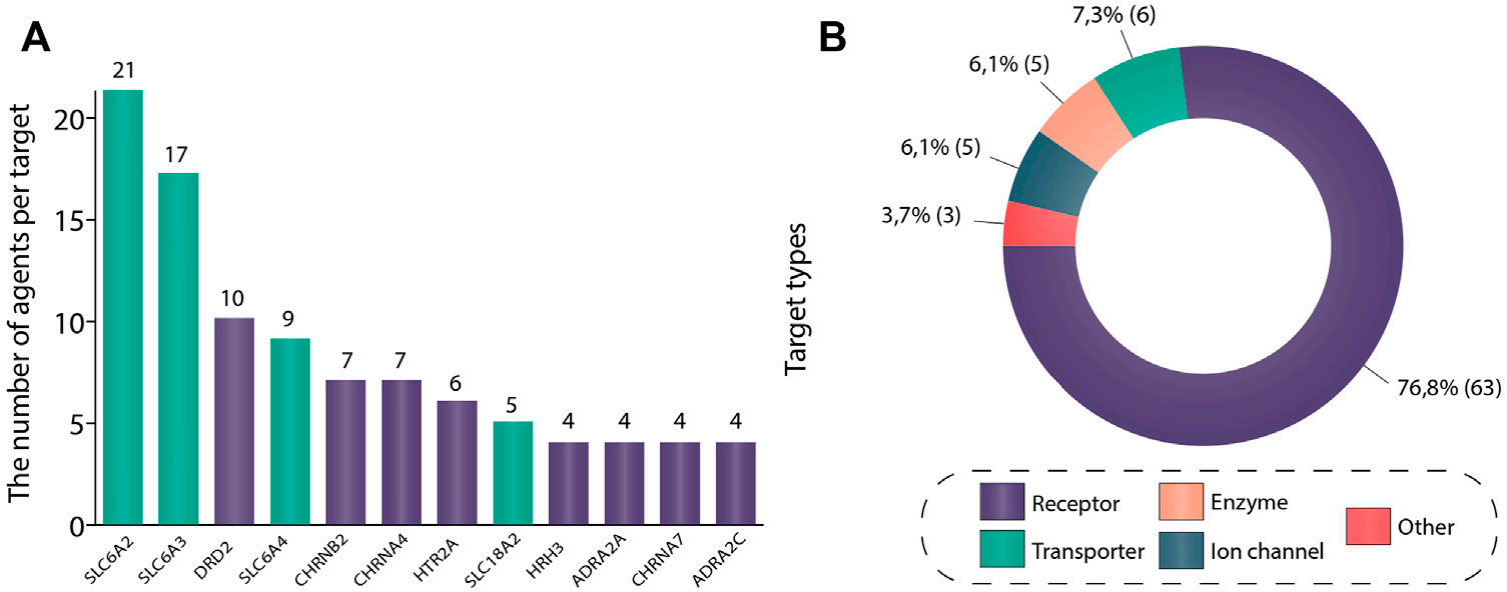
**(A)** Most common drug targets in ADHD trials. This figure shows targets with the highest number of unique agents drugging the corresponding target in ADHD clinical trials. If a drug had multiple targets, all of these targets were included in the analysis. Protein names have been used to distinguish targets. In total, 12 most common drug targets were included in the figure: sodium-dependent noradrenaline transporter (SLC6A2), sodium-dependent dopamine transporter (SLC6A2), D (2) dopamine receptor (DRD2), sodium-dependent serotonin transporter (SLC6A2), neuronal acetylcholine receptor subunit alpha-4 (CHRNB2), neuronal acetylcholine receptor subunit beta-2 (CHRNA4), 5-hydroxytryptamine receptor 2A (HTR2A), synaptic vesicular amine transporter (SLC18A2), histamine H3 receptor (HRH3), alpha-2A adrenergic receptor (ADRA2A), neuronal acetylcholine receptor subunit alpha-7 (CHRNA7) and alpha-2C adrenergic receptor (ADRA2C). (B) Drug target types in ADHD studies. All data regarding drug target type was collected from go.drugbank.com, genome. jp, and uniprot.org. In order to construct this figure, all unique gene names used in the analysis (82 in total) were taken and then transformed into protein names. Then corresponding proteins have been classified according to four target types: receptor, enzyme, ion channel, transporter. For the gene names S100B (S100 Calcium Binding Protein **(B)**, CARTPT (Cocaine- and amphetamine-regulated transcript protein), and M (Matrix protein 2) no information about the protein type was found, and thus they were placed in group “other”. The data is due 4 January 2021.

We investigated the drug targets associated with ADHD. Figure 2A depicts the most common drug targets that were used in ADHD trials: sodium-dependent noradrenaline transporter (21 agents), sodium-dependent dopamine transporter (17 agents), D (2) dopamine receptor (10 agents), sodium-dependent serotonin transporter (9 agents), neuronal acetylcholine receptor subunit alpha-4 (7 agents), neuronal acetylcholine receptor subunit beta-2 (7 agents), 5-hydroxytryptamine receptor 2A (6 agents), synaptic vesicular amine transporter (5 agents), neuronal acetylcholine receptor subunit alpha-7 (4 agents), alpha-2C adrenergic receptor (4 agents), alpha-2A adrenergic receptor (4 agents), and histamine H3 receptor (4 agents). Thus, it can be noted that the predominant number of agents act on sodium-dependent noradrenaline transporter, sodium-dependent dopamine transporter, and sodium-dependent serotonin transporter (gene names: SLC6A2, SLC6A3, SLC6A4, respectively). As patients with ADHD have abnormalities in levels of the corresponding catecholamines, pharmacological agents, acting like reuptake inhibitors, regulate noradrenaline, dopamine, and serotonin neurotransmission and normalize their levels in the brain. It is important to mention that the main class of drugs, approved for ADHD treatment (stimulants) acts primarily on these targets. Amphetamine-type stimulants (also approved for ADHD), in addition to previously mentioned targets, have an effect on synaptic vesicular amine transporter (gene name: SLC18A2), which is a vesicular protein responsible for the accumulation of monoamines (Toren et al., 2005). And for patients who cannot be managed with stimulants, there are alpha-2 adrenergic agonists. This class of drugs targets Alpha-2A adrenergic receptors (gene names: ADRA2A, ADRA2C), which subsequently results in the facilitation of dopamine and noradrenaline neurotransmission. An Alpha-2 agonist medication may be a good option for patients who can’t tolerate stimulant’s side effects, such as insomnia, loss of appetite, significant growth suppression, irritability, tic development or worsening. Additionally, stimulants are contraindicated in patients with tic disorders, heart issues (e.g., congenital heart defects, genetically mediated heightened risk for sudden cardiac death, unstable hypertension or coronary artery disease) or those who are at risk for stimulant abuse or misuse. Sometimes, alpha-2 adrenergic agonists are prescribed along with a stimulant when the stimulant does not work well enough on its own (Cinnamon Bidwell et al., 2010; Hirota et al., 2014). At the moment, most of drugs that are approved for ADHD act *via* enhancing monoamines level in the brain, targeting monoamine transporters (Table 1).

**TABLE 1:** Drugs approved for ADHD and other indications encountered in clinical trials.

Agent name	Agent type	Agent class	Approval overall	Approval ADHD	Targets	First approved indication
Memantine hydrochloride	Small molecule	Dementia therapeutic agent	2003		GRIN1; GRIN2A; GRIN2B; GRIN2C; GRIN2D; GRIN3A; GRIN3B	Dementia of the Alzheimer’s type
Methylphenidate hydrochloride	Small molecule	CNS stimulant	1955	1955	SLC6A3	ADHD
Methylphenidate transdermal system	Small molecule	CNS stimulant	2006	2006	SLC6A3	ADHD
Lisdexamfetamine dimesylate	Small molecule	CNS stimulant	2007	2007	SLC6A2; SLC6A3	ADHD
Viloxazine	Small molecule	Selective noradrenaline reuptake inhibitor	2021	2021	SLC6A2	ADHD
Amiloride hydrochloride	Small molecule	Diuretic	1981		SCNN1A; SCNN1B; SCNN1G; SCNN1D	Congestive heart failure; Hypertension
Amphetamine sulfate	Small molecule	CNS stimulant	1984	1984	SLC18A2; SLC6A2; SLC6A3; CARTPT; TAAR1; VMAT2; MAOA; MAOB	ADHD
Dextroamphetamine sulfate	Small molecule	CNS stimulant	1976	1976	SLC18A2; SLC6A2; SLC6A3	ADHD
Dexmethylphenidate hydrochloride	Small molecule	CNS stimulant	2001	2001	SLC6A3; SLC6A2	ADHD
Mixed amphetamine salts	Small molecule	CNS stimulant	2001	2001	SLC18A2; SLC6A2; SLC6A3	ADHD
Atomoxetine	Small molecule	Selective noradrenaline reuptake inhibitor	2002	2002	SLC6A2	ADHD
Guanfacine hydrochloride	Small molecule	Alpha2 adrenergic receptor agonist	1986	2009	ADRA2A	Hypertension
Clonidine hydrochloride	Small molecule	Alpha2 adrenergic receptor agonist	1974	2010	ADRA2A; ADRA2B; ADRA2C	Hypertension
Molindone hydrochloride	Small molecule	Typical antipsychotic	1974		DRD2	Schizophrenia
Modafinil	Small molecule	CNS stimulant	1998		SLC6A3	Narcolepsy; OSA; SWD
Galantamine hydrobromide	Small molecule	Dementia therapeutic agent	2001		ACHE; CHRNA7	Dementia of the Alzheimer’s type
SDX CL/d-MPH Cl	Small molecule	CNS stimulant	2021	2021	SLC6A3; SLC6A2	ADHD
Quetiapine	Small molecule	Atypical antipsychotic	1997		HTR2A; DRD2	Schizophrenia
Naltrexone hydrochloride	Small molecule	Opiate antagonist	1984		OPRD1; OPRM1; OPRK1; SIGMAR1	Opioid dependence
Ramelteon	Small molecule	Sedative-hypnotic	2005		MTNR1A; MTNR1B	Insomnia
Amantadine	Small molecule	Antiparkinson agent	1966		M; GRIN3A; DRD2	Parkinson’s disease
Bupropion hydrochloride	Small molecule	Antidepressant	1985		SLC6A2; SLC6A3	Major depressive disorder
Vortioxetine	Small molecule	Antidepressant	2013		SLC6A4; HTR3A; HTR7; HTR1B; HTR1A	Major depressive disorder
Aripiprazole	Small molecule	Atypical antipsychotic	2002		DRD2; HTR2A	Schizophrenia
Mazindol	Small molecule	Anoretic	1973		SLC6A2; SLC6A3; SLC6A4	Duchenne muscular dystrophy
Varenicline	Small molecule	Smoking cessation aid	2006		CHRNA4	Smoking addiction
Droxidopa	Small molecule	Antiparkinson agent	2014		ADRA1A; ADRA1B; ADRA1D; ADRA2A; ADRA2B; ADRA2C; ADRB1; ADRB2; ADRB3	Neurogenic orthostatic hypotension
Carbidopa	Small molecule	Antiparkinson agent	2014		DDC	Parkinson’s disease
Duloxetine hydrochloride	Small molecule	Antidepressant	2004		SLC6A4; SLC6A2	MDD; GAD; DPNP; Fibromyalgia; CMP
Zolpidem tartrate	Small molecule	Sedative-hypnotic	1992		GABRA1	Insomnia
Eszopiclone	Small molecule	Sedative-hypnotic	2004		GABRA1; GABRA2; GABRA3; GABRA4; GABRA5; GABRA6; GABRB1; GABRB2; GABRB3; GABRD; GABRE; GABRG1; GABRG2; GABRG3; GABRP; GABRQ	Insomnia
Buspirone hydrochloride	Small molecule	Anxiolytic agent	1986		HTR1A; DRD2	Anxiety disorders
Valproate sodium	Small molecule	Anticonvulsant agent	1996		HDAC9	Complex partial seizures; Simple and complex absence seizures
Risperidone	Small molecule	Atypical antipsychotic	1993		DRD2; HTR2A	Schizophrenia
Brexpiprazole	Small molecule	Atypical antipsychotic	2015		HTR1A; DRD2; HTR2A; ADRA2C; ADRA1B	MDD; Schizophrenia
Divalproex sodium	Small molecule	Anticonvulsant agent	1983		HDAC9	Complex partial seizures; Simple and complex absence seizures
Olanzapine	Small molecule	Atypical antipsychotic	1996		HTR2A; DRD2	Schizophrenia; BD-I; Agitation associated with schizophrenia/BD-I
Oxytocin	Biotech	Uterotonic agent	1980		OXTR	Initiation or improvement of uterine contractions
Fluvoxamine maleate	Small molecule	Antidepressant	1994		SLC6A4	OCD

This table presents information about drug agents approved either for ADHD or other indications that appeared in clinical trials on ADHD. All data regarding agent name, agent type, agent class, years of approval overall and for ADHD, targets, and first approved indication was taken directly from go.drugbank.com, genome. jp, and fda.gov. Gene names were used for the indication of targets. For a list of all protein names, see Supplementary Material S5 (box). Data is due 4 January 2021. Abbreviations: CNS, central nervous system; ADHD, attention deficit hyperactivity disorder.

D (2) dopamine receptor, 5-hydroxytryptamine receptor 2A (gene names: DRD2, HTR2A, respectively) are the key targets of antipsychotics (Aringhieri et al., 2018; Kondej et al., 2018). All members of this class encountered in our analysis have been approved for the treatment of schizophrenia, and none of these agents have been approved for ADHD so far. Another class encountered in trials was represented by dementia therapeutic agents acting on neuronal acetylcholine receptor subunit alpha-4, neuronal acetylcholine receptor subunit beta-2, neuronal acetylcholine receptor subunit alpha-7 (gene names: CHRNA4, CHRNB2, CHRNA7, respectively). Similarly, there are no drugs targeting these receptors that have been approved for ADHD. Nevertheless, all of these targets happened to be rather common in our analysis. Apparently, high costs, low success rates, and complexity of discovering and developing a completely new drug drive the field toward drug repurposing, which may be a more effective and lucrative endeavor and aligns with a global similar growing trend in drug discovery (Pushpakom et al., 2019; Jourdan et al., 2020; Hegvik et al., 2021).

Histamine receptor H3 (gene name: HRH3) is another promising target. Several studies have shown histamine to exert a significant modulatory effect on different brain functions, such as arousal, control of pituitary hormone secretion, cognitive functions, motivation, goal-directed behavior, memory, sleep-wake cycle (Nuutinen and Panula, 2010; Passani et al., 2014), and be involved in diverse behavior and neuropsychiatric diseases (Nieto-Alamilla et al., 2016; Hu and Chen, 2017). To date, it is considered that genetic and pharmacological targeting of histamine receptor H3 alters the level of aggression, circadian rhythms, anxiety, memory, and social behavior and has therapeutic effects on cognitive symptoms in brain disorders (Schneider et al., 2014; Sadek et al., 2016; Reichmann et al., 2020). Thus, all these results point to the potential of histamine receptor H3 modulation as a novel treatment of psychiatric disorders, including ADHD. However, the clinical outcomes regarding medications drugging HRH3 for the treatment of ADHD are somewhat discouraging. Three out of four pharmacological agents drugging this target in our analysis (MK-0249, Bavisant, PF-03654746) reached phase 2 clinical trials where they did not display significant effectiveness in reducing ADHD symptoms (Brioni et al., 2011; Herring et al., 2012; Weisler et al., 2012). The remaining drug (Betahistine) was safe, well-tolerated, and showed no serious adverse events in the phase 1 clinical trial (Moorthy et al., 2015). However, no subsequent studies of this agent in ADHD patients have been initiated since.

In Figure 2B the targets have been simplified to target types based on the following categories: receptor (63, 77%), transporter (6, 7%), enzyme (5, 6%), ion-channel (5, 6%), and protein (3, 4%). Figure 2B reflects a strong quantitative dominance of receptor targets. Receptor and transporter target types jointly represent more than 80% of all drug target types in the included trials. These trends partially correspond to overall trends in drug discovery, where receptors also represent the main drugged class (Rask-Andersen et al., 2011).

## Overall trends in ADHD clinical trials

**FIGURE 3:**
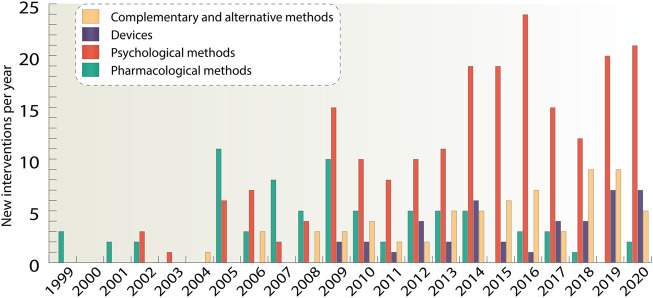
For the four main classes of interventions such as pharmacological interventions, devices, psychological and complementary and alternative interventions, unique new agents and methods per year were taken. If a study had several interventions, all of them were considered separately. The data is due 4 January 2021.

We see upward trends in the number of devices, psychological, as well as complementary and alternative methods, while the number of drug agents fell sharply from 11 agents in 2005 to one agent in 2018 (Figure 3). It can be noticed that the number of psychological methods significantly prevails over the number of all other methods. The largest amount of new interventions can be observed in 2019 and 2020 and it made up 36 interventions per year. The first appearance of a drug agent in clinical trials took place in 1999 as the database started, first psychological methods appeared in 2002, complementary and alternative methods in 2004, whereas devices were first studied only in 2009. Overall, while the number of new non-pharmacological therapies is growing rapidly, drugs do not have such a great diversity of unique agents, so unique non-pharmacological methods exceeded pharmacological ones by more than three times.

**FIGURE 4:**
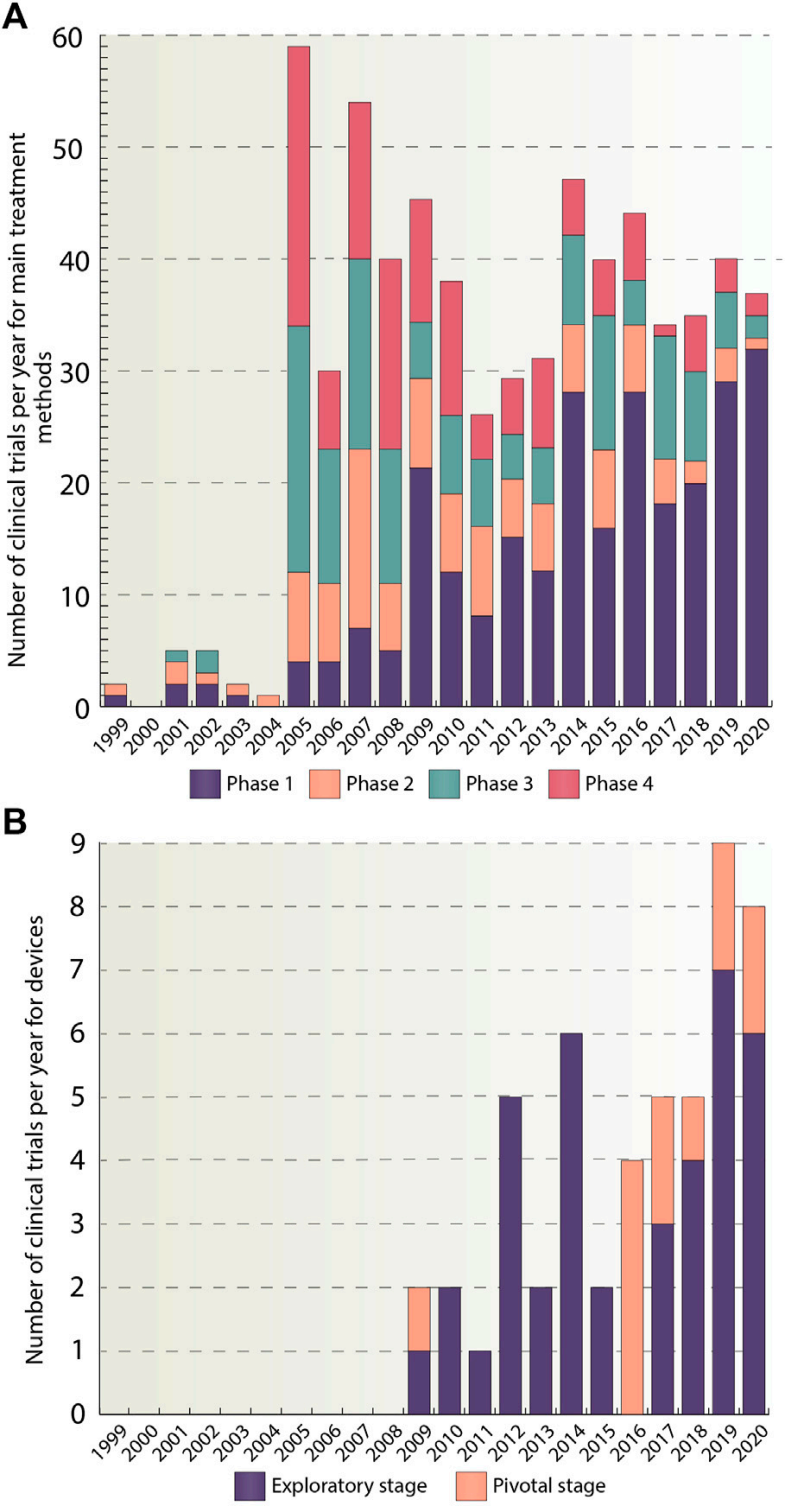
**(A)** Number of clinical trials per year divided by phases. This figure provides a graphical representation of the number of studies per year for pharmacological, psychological and complementary and alternative methods, categorized by a trial phase for the whole analyzed period (1999–2020). In total, 644 studies were included in the analysis. **(B)** Number of clinical trials per year divided by stages. For devices, division by stages was used. Stages have been classified as follows: traditional feasibility, first-in-human, and early feasibility stages have been classified as exploratory stage, the pivotal stage has been left as is. This figure shows the number of device studies for the time period from 1999 to 2020. In total, 51 studies were included. The data is due 4 January 2021.

Our next step was to analyze phase distributions of the trials per year. Figure 4A provides quantitative information about how many studies have been conducted every year since 1999 and what changes occurred in the number of studies within each phase during 1999–2021. It could be seen that the number of trials increased sharply in 2005 and then was approximately at the same level with a slight decrease in 2010–2013 and 2017–2018 time periods. An intermittent increase in the number of clinical trials presented in phase 1 can be also observed. The peak for phase 1 trials was reached in 2020 and amounted to 32 studies. In the second phase, in turn, there was a rise in the number of trials in 2007 (16 trials) followed by their gradual decrease until 2020. In the third and fourth phases, the maximum was reached in 2005 and amounted to 22 and 25 studies, respectively, and then a noticeable decline in their number was observed. The growth in phase 1 trials indicates that more and more therapies are being developed for ADHD treatment. Many of them, however, fail to transit to the subsequent phases of clinical trials, which indicates a low success rate of the therapies.

Figure 4B depicts yearly stage distributions of the clinical trials with medical devices. It should be noted that the first appearance of device trials occurred only in 2009 and amounted to two studies. And 10 years later, in 2019, the number of clinical trials reached its peak and amounted to nine studies. The majority of studies belong to exploratory stage, 39 out of 51, while the remaining ones are in pivotal stage group. The reason for such a large number of trials in exploratory stage may be due to the fact, that devices are a relatively new method of ADHD treatment and therefore only small number of devices have managed to transit to the pivotal stage or got the FDA approval.

## Quantitative trends for phase one and exploratory stage clinical trials

**FIGURE 5:**
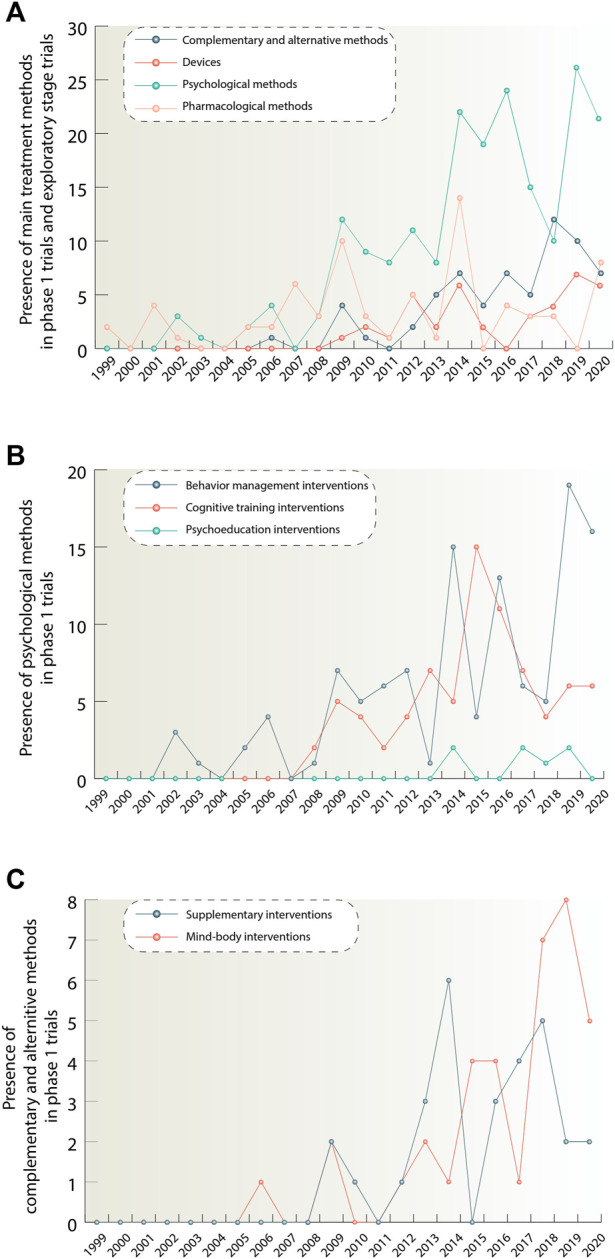
**(A)** Figure 5A illustrates quantitative trends for pharmacological, psychological, complementary and alternative methods and devices. It shows the number of phase 1 studies per year for each method, exept devices, starting from 1999. In oder to show quantitative trends for the device group, studies in exploratory stage were taken. Traditional feasibility, first-in-human, and early feasibility studies have been classified as exploratory stage studies. If there was a combination of interventions in the study, each intervention in this combination was considered separately. **(B)** Figure 5B depicts in more detail quantitative trends for the psychological group after its division into smaller subclasses: behavior management, cognitive training and psychoeducation. **(C)** Figure 5C provides information about quantitative trends distribution for complementary and alternative methods class when divided into supplementary and mind-body intervention groups. The data is due 4 January 2021.

We sought to explore what kinds of therapies demonstrate the increase in phase 1 clinical trials. Figure 5A allows for an overview of trends for phase 1 trials of different types of interventions. For the device group the term « Exploratory stage» was used, instead of «Phase 1». It is worth noting that there is a marked dominance of psychological methods among phase 1 interventions and if we look at the trends over time we can see that the number of these interventions increased dramatically during the years 2008–2020. We divide psychological methods into subclasses (behavior management interventions, cognitive training interventions, psychoeducation interventions) in order to understand which of them underlie this growth to a greater extent (Figure 5B). It is seen that the bulk of psychological interventions are behavior management therapies, which amount to 115 studies during the analyzed period. The next largest group is cognitive training interventions accounting for 78 studies. And then the smallest group–psychoeducation with seven studies.

Also, based on the data presented in Figure 5A, we can highlight the growth of such classes as devices and complementary and alternative methods. According to Figure 5C, it can be concluded, that the complementary and alternative methods group consisting of supplementary and mind and body interventions showed 36 interventions in total, while mind and body interventions demonstrated the highest growth for this treatment direction. For the device class, it was not possible to allocate equivalent subclasses.

## Drug classes, trials, and promising drug candidates

**FIGURE 6:**
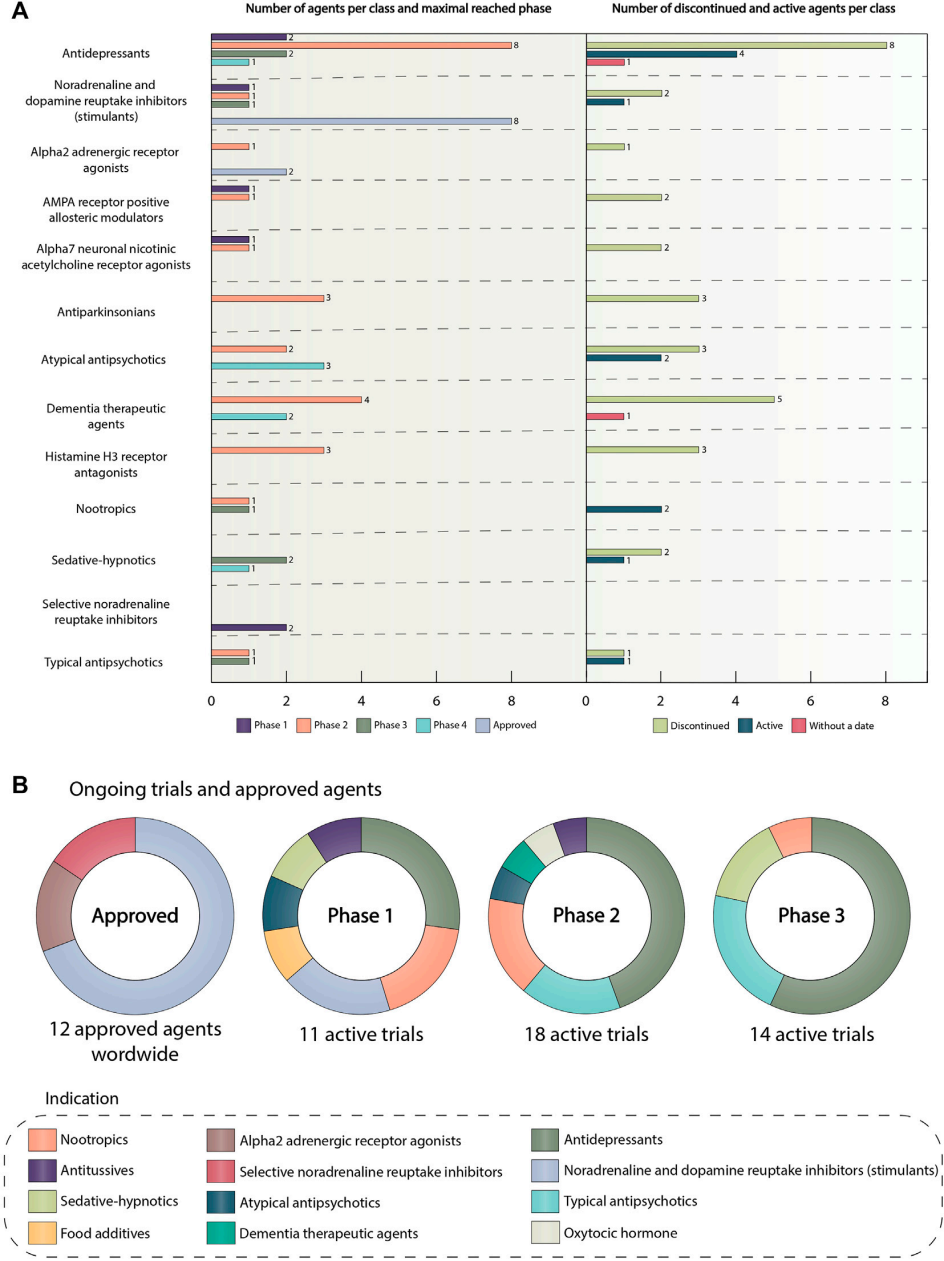
**(A)** Number of agents per category and maximal reached phase. The number of discontinued and active agents per category. This graph presents information about unique agents and their maximal reached phase in the following classes: antidepressants, central nervous system stimulants, alpha2 adrenergic receptor agonists, AMPA receptor positive allosteric modulators, alpha7 neuronal nicotinic acetylcholine receptor agonists, antiparkinsonian, atypical antipsychotics, dementia therapeutic agents, histamine H3 receptor antagonists, nootropics, sedative-hypnotics, selective noradrenaline reuptake inhibitors, typical antipsychotics. The colors of the bars show the maximal reached phase or approval status. The right side of the graph depicts the number of active or discontinued agents in the classes presented on the left side. The drug was classified as active if it was not approved and there were clinical trials on it after 2018. The drug was classified as discontinued if it was not approved, and there were no active clinical trials on it after 2018. If there was no completion date for the agent, it was placed in the “without a date” category. The length of the bars is proportional to the number of agents. The colors of the bars show the agent category. **(B)** Ongoing trials and approved agents The pie charts show the distribution of drug classes in ongoing clinical trials and among approved ADHD agents. The colors of the slices correspond to the pharmacological classes. Trials have been sub-categorized by phase. The data is due 4 January 2021. Abbreviations: ADHD, attention deficit hyperactivity disorder; α-amino-3-hydroxy-5-methyl-4-isoxazolepropionic acid receptor, AMPA receptor.

Figure 6A depicts pharmacological classes of drugs encountered in clinical trials, which had at least two unique representatives. In other words, at least two drugs of one pharmacological class with different INNs should have been investigated in clinical trials in order to include that class in this figure. List of all pharmacological agents encountered in clinical trials can be observed in Supplementary Material S6. It can be concluded that antidepressants and central nervous system stimulants are classes with the highest number of unique agents, most of which are in phase 2 trials at the moment. The stimulants group, in turn, contains the highest number of approved agents - eight agents out of 12 approved for ADHD overall. The number of unique entities in other groups ranges from two to six agents per class.

Surprisingly, we can notice that the overwhelming majority of agents are discontinued, 31 out of 44 agents in total for all of the classes taken for analysis. The main reason for drug discontinuation was lack of efficacy or safety (e.g., bradanicline, vortioxetine, bavisant, PF-03654746, NS-2359, AZD1446) (Learned et al., 2012; Haig et al., 2014; Pozzi et al., 2020). It is worth noting, that all of these agents did not succeed in phase 2 clinical trials. Furthermore, phase 3 study, assessing the effectiveness of metadoxine in ADHD patients was terminated by the FDA due to possible adverse events (Di Miceli and Gronier, 2018; US National Library of Medicine, 2022a). In addition, modafinil–stimulant, initially approved by the FDA as a wakefulness-promoting agent failed to become a treatment option for ADHD patients due to safety concerns (Heal et al., 2013). For some medications, no results were published (e.g., CX717) (Pozzi et al., 2020). Thus, only 10 unique agents are currently active, and the most promising class in this group is represented by antidepressants accounting for four agents. For two agents there was no completion date in the corresponding trials, thus we assigned them to the «without a date » category. The most interesting and promising active drug candidates included PDC-1421, centanafadine, ADAIR, dasotraline, and tipepidine.

PDC-1421—an extract of Polygala tenuifolia, which is an active ingredient of ABV-1505, a plant-based drug targeting ADHD symptoms in adults, developed by BioLite, Inc. PDC-1421 is an antidepressant, which functions as norepinephrine reuptake inhibitor (PDC-1421 – AdisInsight, 2022). The phase 2 part 1 study, investigating PDC-1421 for treating ADHD demonstrated the therapeutic value of this drug. It was found that the PDC-1421 capsule was safe, well-tolerated, and efficacious in six adult patients with ADHD (US National Library of Medicine, 2022b). Now this drug is tested on a larger cohort to determine the effective doses and treatment period of PDC-1421 capsule in ADHD patients. (US National Library of Medicine, 2022b). Centanafadine is a triple monoamine reuptake inhibitor, which demonstrates the highest potency for norepinephrine and dopamine. Phase 2 studies indicated the therapeutic effectiveness of this drug (Wigal et al., 2020). Phase 3 randomized controlled trails demonstrated centanafadine to be safe, well-tolerated and effective in addressing the core symptoms of ADHD in adults (Adler et al., 2022) Currently, centanafadine is under investigation in several clinical trials assessing its efficacy and safety in children and adolescents with ADHD (e.g., NCT05279313, NCT05428033, NCT05257265). Tipepidine is a small-molecule non-narcotic antitussive drug that has been used in children in Japan since 1959. It is assumed that the pharmacological effect of tipepidine on ADHD symptoms is due to the inhibition of G protein-coupled inwardly rectifying potassium (GIRK)-channel currents with the subsequent regulation and balancing of monoamines level in the brain (Hashimoto and Sasaki, 2015) Tipepidine showed some promising results, regarding improvement of ADHD symptoms in several phase 2 clinical trials, however, more trials with the big sample are needed to validate the therapeutic benefits of this agent (Pozzi et al., 2020). At the moment, no subsequent studies have been initiated since. Dasotraline is a serotonin-norepinephrine-dopamine reuptake inhibitor, which in structure is a stereoisomer of an active metabolite of sertraline, a selective serotonin reuptake inhibitor (SSRI) antidepressant (Uphar/Bps Guide to Pharmacology, 2022). Dasotraline was developed by Sunovion Pharmaceuticals Inc. and considered to be a rather promising novel therapeutic agent for ADHD treatment, also offering lower abuse potential compared to conventional therapies (Vandana and Arnold, 2019). Like other drugs presented above, dasotraline was recently active in clinical trials. However, due to a lack of clinical data to secure approval, the company decided not to pursue any further development of this agent in May 2020 (Sunovion Discontinues Dasotraline Program, 2020).

As misuse of stimulant medications is a serious ongoing public health issue (Clemow and Walker, 2014). ADAIR (Abuse Deterrent Amphetamine Immediate Release) developed by Vallon Pharmaceuticals Inc. is a novel oral formulation of immediate release dextroamphetamine designed specifically to limit abuse of this drug by snorting or injecting (Vallon Pharmaceuticals Inc, 2021). The safety profile of ADAIR is considered to be no different from the corresponding stimulants, but appears to be less desirable to recreational drug abusers when snorted. In March 2022, Vallon Pharmaceuticals reported main results obtained in its SEAL study, evaluating abuse liability, pharmacokinetics, safety and tolerability of ADAIR. Despite the fact that this study did not meet its primary endpoint, which was E_max_ drug liking, it showed some favorable outcomes on secondary endpoints, such as overall drug liking and willingness to take drug again, additional endpoints of E_max_ overall drug liking and E_max_ take drug again, and other exploratory pharmacokinetic and pharmacodynamic endpoints. These results support further investigation of this drug and if approved, ADAIR is expected to be the first abuse-deterrent, immediate-release formulation of dextroamphetamine (Vallon Pharmaceuticals Inc, 2022; US National Library of Medicine, 2021a).

## Ongoing trials and approved agents

The first pie chart in Figure 6B shows that there are 12 agents approved for ADHD, including the transdermal system (methylphenidate transdermal system, which is norepinephrine and dopamine reuptake inhibitor). In the alpha2 adrenergic receptor agonists group, there are guanfacine hydrochloride and clonidine hydrochloride. Representatives of the selective noradrenaline reuptake inhibitor group are atomoxetine and approved in 2021 drug–viloxazine (FDA, 2021a). Initially, Viloxazine or SPN-812 had been developed and approved in the UK for the treatment of depression, but later it was withdrawn from the market because of business reasons unrelated to the therapeutic effectiveness of the drug or its safety (Faison et al., 2021). The main action of viloxazine is to increase the brain norepinephrine concentrations, but it is also considered to facilitate the neurotransmission of serotonin, and, to a lesser extent, dopamine (Yu et al., 2020; Findling et al., 2021). Patients with ADHD notoriously have depleted levels of these catecholamines (Prince, 2008), so, viloxazine has become the perfect candidate for ADHD treatment and showed significant results in several randomized clinical trials (Findling et al., 2021). Such pharmacological agents as lisdexamfetamine dimesylate, methylphenidate hydrochloride, amphetamine sulfate, dexmethylphenidate hydrochloride, dextroamphetamine sulfate, mixed amphetamine salts, a combination of dexmethylphenidate hydrochloride and serdexmethylphinidate chloride represent noradrenaline and dopamine reuptake inhibitor class. It is worth noting that, previously known as KP415, a combination of serdexmethylphenidate, which is a prodrug of dexmethylphenidate, with immediate-release dexmethylphenidate also received its approval in 2021 and is currently marketed the trademark AZSTARYS (FDA, 2021b). Dexmethylphenidate now is used as a central nervous system stimulant, that increases extracellular levels of dopamine and norepinephrine in the CNS, which leads to alleviation of ADHD symptoms (FDA, 2021c). For more information about agents approved for ADHD and other indications encountered in clinical trials refer to Table 1.

The next three pie charts summarize all active trials divided by classes and phases. It can be observed that the prevailing class in all of these phases is antidepressants. In the first phase, the number of studies containing antidepressants is three, for the second and third phase, the quantity is eight studies per phase. The total amount of active pharmacological interventions is 41 (12 active trials in the first phase, 18 trials in the second, and 14 in the third).

## Devices as a novel promising method of ADHD treatment

Our data shows an intriguing slight increase in the number of device studies (Figure 3), starting from 2009. One of the most frequent forms of device treatment that occurred in clinical trials was transcranial direct current stimulation (tDCS) (Supplementary Figure S2). It is a non-invasive, painless method of treatment that uses direct electrical currents to stimulate specific parts of the brain (Brunoni et al., 2012). The stimulation arises by providing a constant weak current through two electrodes (anodal and cathodal) placed over the head, which subsequently leads to modulation of neuronal activity (Thair et al., 2017; Chase et al., 2020). It is assumed that tDCS has a positive effect on executive functions, such as working memory, attention and inhibitory control, which are typically impaired in people with ADHD (Cosmo et al., 2020). Also, in patients affected by this disease, tDCS is considered to alleviate symptoms of impulsivity (Allenby et al., 2018). Despite the fact that this form of device treatment is rather promising, no device representing this class has been approved by the FDA for ADHD treatment yet.

The next most popular device class that appeared in clinical trials was repetitive transcranial magnetic stimulation (rTMS) that was cleared by the FDA for the treatment of major depression and obsessive compulsive disorder (Supplementary Figure S2). It is a multisession and non-invasive treatment method, where a changing magnetic field is used to cause electric current, which subsequently generates the stimulation of brain neurons (Iglesias, 2020). Hence, brain activity changes, and it can lead to the symptom elimination of various psychiatric disorders such as depression, schizophrenia, Tourette’s syndrome, and other (Ziemann et al., 1997; Wassermann and Lisanby, 2001). However, this method has somewhat conflicting results regarding its effectiveness in reducing and alleviating the symptoms of ADHD (Wong and Zaman, 2019).

As of 2021, there are at least two devices that received FDA approval for the treatment of ADHD: Monarch eTNS System and EndeavorRx. Monarch eTNS System is the first device-based ADHD treatment, that received clearance from the FDA (FDA, 2019). It is a non-invasive, small, electronic device, which generates electrical signals to provide low-level stimulation to the branches of the trigeminal nerve. And though the exact mechanism of external trigeminal nerve stimulation (eTNS) is still unknown, it is considered to increase activity in brain regions such as anterior cingulate, inferior frontal gyrus, medial and middle frontal gyri known to be important in regulating executive functions that are impaired in patients with ADHD (Cook et al., 2014; Loo et al., 2021). Currently, Monarch eTNS System is used as a monotherapy treatment in ADHD patients ages 7–12 years old under the supervision of a caregiver. EndeavorRx (ProjectEVO, AKL-T01) is a first-of-its-kind game-based digital therapeutic device for children 8–12 years old with ADHD approved by the FDA in June 2020 (FDA, 2020). It was developed to engage children through using high-quality graphics and reward loops and providing feedback on progress and compliance. EndeavorRx is supposed to target those areas of the brain that are responsible for attention function, which leads to improvements in attention, inhibitory control, and working memory that are most affected in patients with ADHD (Davis et al., 2018). In this regard, it can be concluded that devices are promising rapidly developing drug-free alternative for ADHD treatment.

## Physical activity and behavioral tools to treat ADHD

Non-pharmacological methods, in addition to devices, include two big groups: psychological therapies and complementary and alternative therapies. Based on our data, it is clear that the number of complementary and alternative interventions has increased for the past decade, with the largest number of studies in the physical activity (PA) field (Supplementary Figure S3). Aerobic exercise, Treadmill training, Sprint interval training, Baduanjin practice, Taekwondo practice, MOVI KIDS, ImPuls, Equine-assisted activities and therapies are all examples of practices, which have been used as trial interventions to treat patients with ADHD. It is assumed that individuals with ADHD have reduced levels of dopamine and norepinephrine in the brain, which results in poor cognitive performance. Therefore, a possible PA mechanism for alleviating ADHD symptoms may be an increase in the neurotransmission and availability of the catecholamines in brain networks, which consequently can lead to improvements in executive functions (Tomporowski et al., 2008; Wigal et al., 2013). Many studies have shown positive effects on academic performance, behavior problems, motor and emotional control, planning and problem-solving (Sanchez-Lopez et al., 2015; Suarez-Manzano et al., 2018). In some trials, a substantial improvement in anxiety and depression symptoms, aggressive behaviors, and social problems were observed (Zang, 2019). Despite the fact that there is no strong base of evidence regarding the effectiveness of physical exercise, it does not seem unreasonable to think that PA could be a promising additional method for the management of ADHD (Tomporowski et al., 2008; Wigal et al., 2013; Sanchez-Lopez et al., 2015; Suarez-Manzano et al., 2018; Zang, 2019; Perez-Gomez et al., 2020).

The main growth in the field of psychological therapies could be explained by behavior interventions. There are two clinical trials in particular that worth noting: NCT03628781 and NCT04402528. Behavioral therapies happened to be rather effective in eliminating the symptoms of ADHD (Pfiffner and Haack, 2014; Catala-Lopez et al., 2017). However, there are some obstacles, such as the paucity of trained providers, financial and geographic issues, which prevent psychological methods from having the desired effect. Hence, some behavioral tools integrated within Mehealth for ADHD software were developed to eliminate these barriers and abate functional impairment in people with ADHD. Mehealth for ADHD is an evidence-based, secure online technology portal that helps pediatricians to provide better ADHD care and increases rates of behavioral treatment (Epstein et al., 2013). Also, it is a unique tool to improve communication with patients, save time in diagnosis, and optimize ADHD treatment. This therapy has recently completed two clinical trials on 200 participants in total (US National Library of Medicine, 2021b; US National Library of Medicine, 2021c).

## Discussion

This review provides comprehensive quantitative data on pharmacological and non-pharmacological methods for the treatment of ADHD that have reached clinical trials in children, adolescents, and adults, covering 695 studies. Overall, we see that the number of unique non-pharmacological interventions (Supplementary Figure S1) is three-fold higher than the number of unique interventions on pharmacological agents. Psychological interventions have become the dominant class among non-pharmacological methods in terms of the number of clinical trials. Moreover, there is a high number of studies using a different kind of devices and the number of complementary and alternative interventions have also increased over the past decade (Figure 3).

There are 12 drugs approved by the FDA for treatment of ADHD (methylphenidate hydrochloride, methylphenidate transdermal system, lisdexamfetamine dimesylate, amphetamine sulfate, dexmethylphenidate hydrochloride, dextroamphetamine sulfate, mixed amphetamine salts, dexmethylphenidate hydrochloride/serdexmethylphenidate chloride, atomoxetine, guanfacine hydrochloride, clonidine hydrochloride, viloxazine). In addition to approved agents, in the classes that have at least two unique representatives, there are five novel emerging drugs (ADAIR, PDC-1421, centanafadine, OPC-64005, prospecta) being tested in the clinical trials with one, three, and one agent that have reached phases 1, 2, and 3, respectively. Also, there are three repurposed drugs (risperidone, brexpiprazole, molindone hydrochloride) that have been earlier approved for the treatment of other indications and now are tested in clinical trials for ADHD. And two drugs (fasoracetam and dasotraline) with a suspended clinical development. The largest class of ADHD pharmacological treatment are central nervous system stimulants (e.g., methylphenidate hydrochloride, lisdexamfetamine dimesylate, amphetamine sulfate, mixed amphetamine salts) that act predominantly by regulating the brain levels of monoamines (mostly dopamine and norepinephrine), drugging the sodium-dependent noradrenaline transporter and sodium-dependent dopamine transporter. Antidepressants are the class with the highest number of unique agents in clinical trials (e.g., bupropion hydrochloride, vortioxetine, duloxetine hydrochloride, edivoxetine, reboxetine, fluvoxamine maleate, PDC-1421, dasotraline, ampreloxetine). Antidepressants could be prescribed to treat ADHD in patients who did not respond or showed intolerance to stimulants since antidepressants have a similar mechanism of action and drug the same targets (e.g., sodium-dependent noradrenaline transporter, sodium-dependent dopamine transporter) as psychostimulants. However, the FDA has not specifically approved these medications for this.

We see that there is a clear increase in the number of behavior management trials and cognitive training trials (Supplementary Figure S4). The success of behavioral therapies (e.g. behavior parent training, school-based interventions, peer-based interventions) in eliminating the symptoms of ADHD may be largely due to the fact that the treatment begins mainly from childhood with the help of parents/teachers/caregivers and children can use the acquired skills to cope with ADHD symptoms throughout their lives. Such therapies are also fairly harmless and could be suggested to be used if the diagnosis is not certain. In addition, there is a wide variety of treatment approaches, targeting different age groups (Young and Amarasinghe, 2010; Shrestha et al., 2020). The effectiveness of cognitive therapies can be related to the ability of such therapies to target the key elements of cognition and intelligence, such as attention, inhibitory control, working memory, planning, and cognitive flexibility that contribute to thinking and learning impaired in patients with ADHD (Veloso et al., 2019).

Overall, the increase in the number of non-pharmacological studies may be related to the fact that interventions are focused on the individuals with ADHD as they grow up and can be modified from childhood to adulthood, exerting influence on the most problematic behavioral symptoms at that stage in time. As children mature, non-pharmacological interventions may be changed according to the developmental needs and circumstances related to the particular individual. For each age category, there are the most preferred methods of non–pharmacological treatment, such as parent training for preschool children or parent training and classroom interventions for school-age individuals (Young and Amarasinghe, 2010). Besides, the process of conducting pharmacological clinical trials is extremely costly (ASPE, 2014; Collier, 2009; Martin et al., 2017), which can also contribute to the growth in the quantity of non-pharmacological trials. Although pharmacological treatments may not always be acceptable or tolerable for all patients. And even despite the fact, that medication may acutely alleviate symptoms of ADHD, support with skill development and other behavioral therapies are still needed (Russell et al., 2022). In addition, there are several non-pharmacological therapies, targeting one particular domain impaired in patients with ADHD, such as working memory and attention trainings, social skills therapies, interventions to improve academic performance.

Despite the fact, that the number of unique pharmacological interventions is not high (Supplementary Figure S1), drugs remain the first-line treatment of ADHD (Vaughan et al., 2012). The current treatment is based on using medications alone or in combinations with behavioral therapies in order to boost dopamine and noradrenaline neurotransmission in the brain. It should be noted that the number of drugs approved for ADHD treatment is not very high (12 unique agents, where most of the representatives are derivatives of methylphenidate hydrochloride and amphetamine sulfate) and act on a limited number of targets (Hegvik et al., 2021). Drug management of ADHD is still a developing field: for instance, a combination of dexmethylphenidate hydrochloride and serdexmethylphenidate (represents central nervous system stimulant class) have been approved for ADHD treatment in 2021 (Clemow and Walker, 2014). Additionally, there are several promising drug candidates (PDC-142, centanafadine, tipepidine) that are under research and development and have been used as trial interventions during the last 3 years period. Moreover, the emergence of new drug formulations (ADAIR), presence of promising targets (HRH3), and desire to overcome such issues as short-term effectiveness, low adherence, and non-tolerability (Pozzi et al., 2020) also contributes to the development of the field.

ADHD treatment can also be provided by using combinations of different methods. As previous studies have shown, the combination of methylphenidate hydrochloride with behavioral therapies had some significant effects on ADHD symptomatology, which perfectly correlates with the data obtained during our analysis, where the predominant number of approved drugs are stimulants (Figure 6B) and the largest number of psychological studies have been conducted in the field of behavior management interventions (Supplementary Figure S4). It should be noted that behavior therapy, with parent training as the most studied psychological intervention, is the only non-pharmacological method that showed statistically significant results in clinical trials, and a combination of behavior therapy with stimulants was more effective than stimulants alone (Catala-Lopez et al., 2017). While the medications work on regulating neurotransmission of monoamines in the brain, the behavioral therapy is more targeted towards specific problem behaviors, such as poor academic performance, bad relationships with relatives and peers, and aggressive behavior. Another combination of methylphenidate hydrochloride with non-stimulant medications, such as atomoxetine, clonidine, and guanfacine also showed very promising results in the reduction of ADHD symptoms. However, more research in the sphere of treatment combinations is needed.

We can conclude that the area of ADHD treatment is gaining increased complexity with high variety of different treatment options, such as drugs, behavior management interventions, cognitive training therapies, supplementary interventions, devices, mind and body interventions. There are several reasons that drive the increased variety of therapies to treat ADHD such as the misuse of stimulant medications, the ability of behavior therapies to target mainly behavior problems without normalizing the level of catecholamines in the brain, financial limitations, and family logistical challenges. Future research is warranted to gain a deeper understanding of the potential effects of non-pharmacological therapies (including devices) to provide a mechanistic explanation on how these interventions (alone or in combination) impact the pathology of ADHD or comorbid symptoms. In addition, it is common that treatment of ADHD begins after the disease has already become detrimental to a person’s life when all characteristic disease symptoms are present. Hence, early diagnosis and prevention of the disorder is likely to become increasingly important area. Lastly, it is also important to investigate how available and future treatment options (including non-pharmacological ones) impact the person’s social life and career longitudinally since these long-term outcome measures are effectively one of the main reasons to treat ADHD in the first place (Harpin et al., 2016).

## Abbreviations

ADAIR, Abuse deterrent amphetamine immediate release; ADHD, Attention-deficit/hyperactivity disorder; ADHD-C, Attention-deficit/hyperactivity disorder combined type; ADHD-H, Attention-deficit/hyperactivity disorder predominantly hyperactive–impulsive type; ADHD-I, Attention-deficit/hyperactivity disorder predominantly inattentive type; CNS, Central nervous system; CBT, Cognitive behavioral therapy; dTMS, Direct transcranial magnetic stimulation; eTNS, External trigeminal nerve stimulation; FDA, U.S. Food and Drug Administration; GIRKs, G protein-coupled inwardly-rectifying potassium channels; GPCRs, G protein-coupled receptors; INN, International nonproprietary name; MRI, Magnetic resonance imaging; PA, Physical activity; PFC, Prefrontal cortex; rTMS, Repetitive transcranial magnetic stimulation; tDCS, Transcranial direct-current stimulation; tRNS, Transcranial random noises stimulation; SSRI, Selective serotonin reuptake inhibitor; VR, Virtual reality.
