# Supplementary material for: Treatment of ADHD: Drugs, psychological therapies, devices, complementary and alternative methods as well as the trends in clinical trials
Source: Front Pharmacol. 2022 Nov 17;13:1066988. doi: 10.3389/fphar.2022.1066988 (PMC9713849; doi:10.3389/fphar.2022.1066988)
Supplement: Supplementary file 1 [file DataSheet1.docx]

Supplementary Material

# Supplementary Data


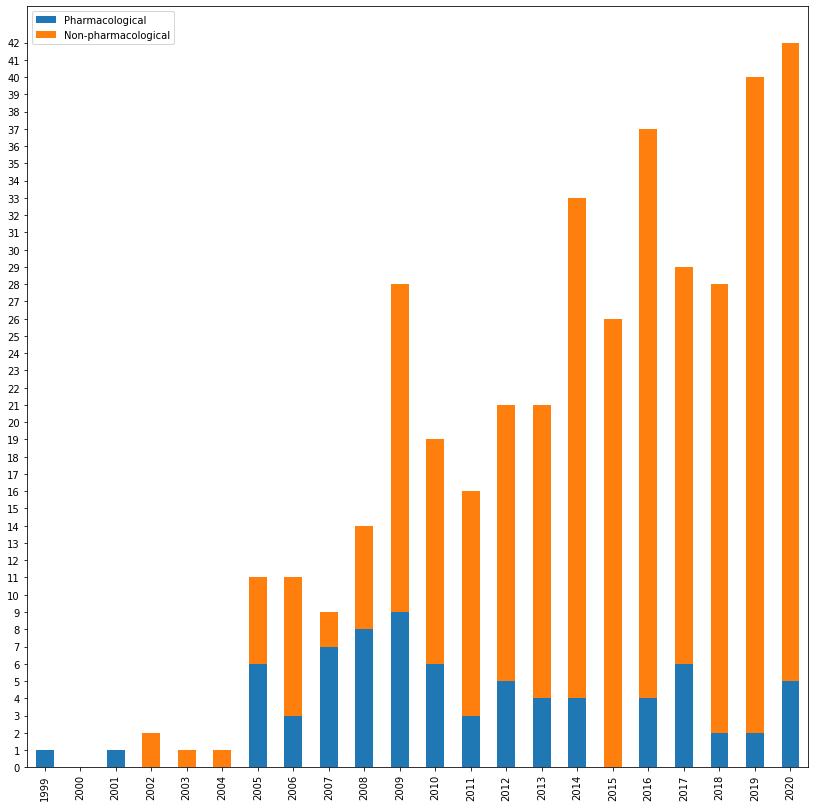


**Figure S1: Number of pharmacological interventions versus non-pharmacological over time.** This figure depicts the quantity of unique pharmacological and non-pharmacological interventions per year for the whole analyzed period (1999 – 2020). The data is due January 4, 2021.

# Supplementary Data


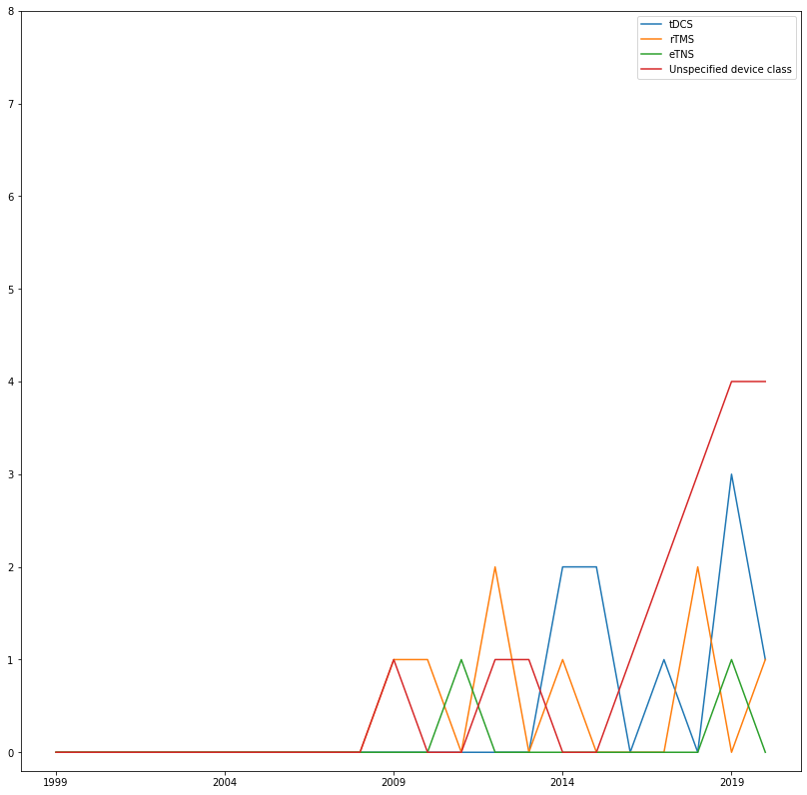


**Figure S2: The most common device classes used in clinical trials.** The figure presents information about the number of unique devices encountered in clinical trials for the most common device classes since 1999. The data is due January 4, 2021. Abbreviations: tDCS, transcranial direct current stimulation; rTMS, repetitive transcranial magnetic stimulation; eTNS, external trigeminal nerve stimulation.

# Supplementary Data


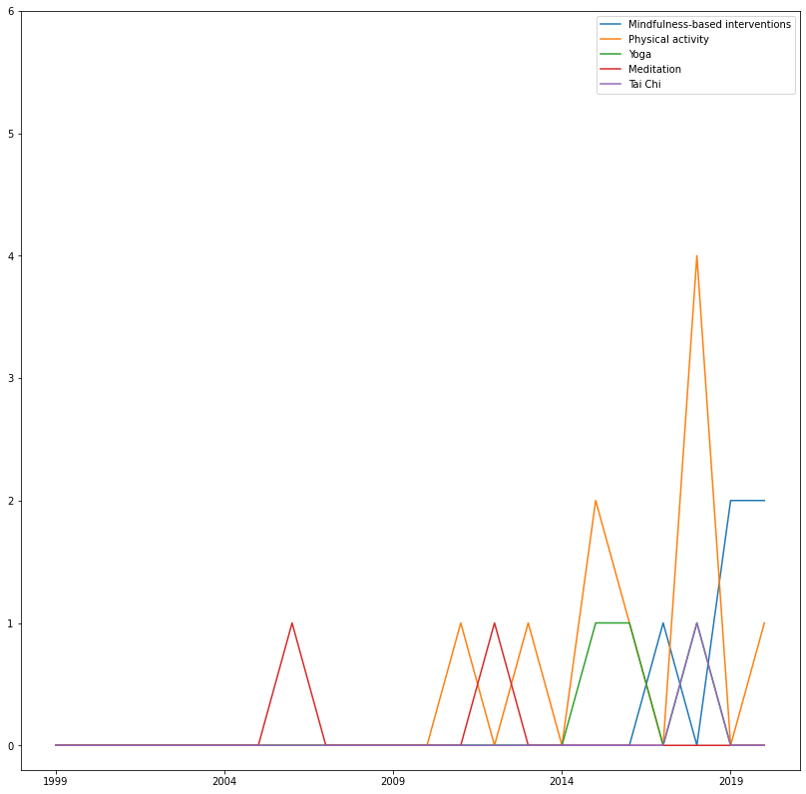


**Figure S3: The most common complementary and alternative therapies used in clinical trials.** This figure shows complementary and alternative therapies with the highest number of clinical trials for the whole analyzed period, starting from 1999. The data is due January 4, 2021.

# Supplementary Data


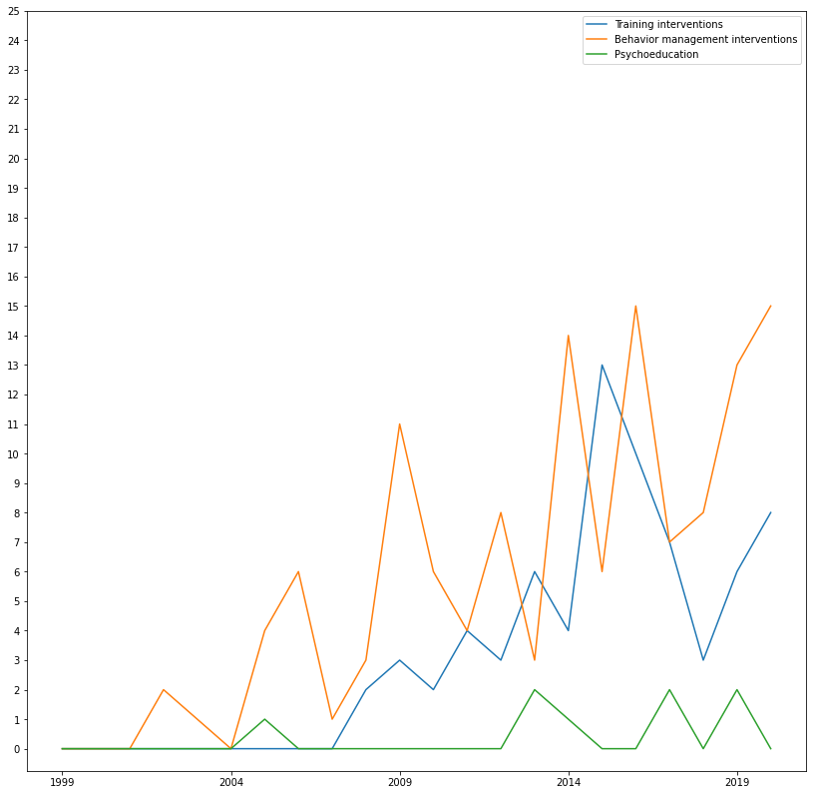


**Figure S4: The number of psychological interventions divided by classes.** The figure provides a graphical representation of the quantity of unique interventions per year in cognitive training, behavior management and psychoeducation groups. The data is due January 4, 2021.

# Supplementary Data

Supplementary information S5 | List of all gene abbreviations included in Table 1

GRIN1, [Glutamate receptor ionotropic, NMDA 1](https://go.drugbank.com/polypeptides/Q05586); GRIN2A, [Glutamate receptor ionotropic, NMDA 2A](https://go.drugbank.com/polypeptides/Q12879); GRIN2B, [Glutamate receptor ionotropic, NMDA 2B](https://go.drugbank.com/polypeptides/Q13224); GRIN2C, [Glutamate receptor ionotropic, NMDA 2C](https://go.drugbank.com/polypeptides/Q14957); GRIN2D, [Glutamate receptor ionotropic, NMDA 2D](https://go.drugbank.com/polypeptides/O15399); GRIN3A, [Glutamate receptor ionotropic, NMDA 3A](https://go.drugbank.com/polypeptides/Q8TCU5); GRIN3B, [Glutamate receptor ionotropic, NMDA 3B](https://go.drugbank.com/polypeptides/O60391); SLC6A2, Sodium-dependent noradrenaline transporter; SLC6A3, Sodium-dependent dopamine transporter; SCNN1A, [Amiloride-sensitive sodium channel subunit alpha](https://go.drugbank.com/drugs/DB00594#BE0000190); SCNN1B, [Amiloride-sensitive sodium channel subunit beta](https://go.drugbank.com/drugs/DB00594#BE0000024); SCNN1G, [Amiloride-sensitive sodium channel subunit gamma](https://go.drugbank.com/drugs/DB00594#BE0000497); SCNN1D, [Amiloride-sensitive sodium channel subunit delta](https://go.drugbank.com/drugs/DB00594#BE0000495); SLC18A2, Synaptic vesicular amine transporter; CARTPT, Cocaine- and amphetamine-regulated transcript protein; TAAR1, Trace amine-associated receptor 1; VMAT2, Synaptic vesicular amine transporter; MAOA, [Amine oxidase [flavin-containing] A](https://go.drugbank.com/polypeptides/P21397); MAOB, [Amine oxidase [flavin-containing] B](https://go.drugbank.com/polypeptides/P27338); ADRA2A, Alpha-2A adrenergic receptor; ADRA2B, Alpha-2B adrenergic receptor; ADRA2C, Alpha-2C adrenergic receptor; DRD2, D(2) dopamine receptor; ACHE, Acetylcholinesterase; CHRNA7, Neuronal acetylcholine receptor subunit alpha-7; HTR2A, 5-hydroxytryptamine receptor 2A; OPRD1, **Delta-type opioid receptor**; OPRM1, **Mu-type opioid receptor**; OPRK1, **Kappa-type opioid receptor**; SIGMAR1, Sigma non-opioid intracellular receptor 1; MTNR1A, **Melatonin receptor type 1A**; MTNR1B, **Melatonin receptor type 1B**; M, **Matrix protein 2**; SLC6A4, Sodium-dependent serotonin transporter; HTR3A, 5-hydroxytryptamine receptor 3A; HTR7, 5-hydroxytryptamine receptor 7; HTR1B, 5-hydroxytryptamine receptor 1B; HTR1A, 5-hydroxytryptamine receptor 1A; CHRNA4, Neuronal acetylcholine receptor subunit alpha-4; ADRA1A, Alpha-1A adrenergic receptor; ADRA1B, Alpha-1B adrenergic receptor; ADRA1D, **Alpha-1D adrenergic receptor**; ADRB1, Beta-1 adrenergic receptor; ADRB2, **Beta-2 adrenergic receptor**; ADRB3, **Beta-3 adrenergic receptor**; GABRA1, Gamma-aminobutyric acid receptor subunit alpha-1; GABRA2, [Gamma-aminobutyric acid receptor subunit alpha-2](https://go.drugbank.com/polypeptides/P47869); GABRA3, Gamma-aminobutyric acid receptor subunit alpha-3; GABRA4, Gamma-aminobutyric acid receptor subunit alpha-4; GABRA5, Gamma-aminobutyric acid receptor subunit alpha-5; GABRA6, Gamma-aminobutyric acid receptor subunit alpha-6; GABRB1, [Gamma-aminobutyric acid receptor subunit beta-1](https://go.drugbank.com/polypeptides/P18505); GABRB2, [Gamma-aminobutyric acid receptor subunit beta-2](https://go.drugbank.com/polypeptides/P47870); GABRB3, [Gamma-aminobutyric acid receptor subunit beta-3](https://go.drugbank.com/polypeptides/P28472); GABRD, [Gamma-aminobutyric acid receptor subunit delta](https://go.drugbank.com/polypeptides/O14764); GABRE, [Gamma-aminobutyric acid receptor subunit epsilon](https://go.drugbank.com/polypeptides/P78334); GABRG1, [Gamma-aminobutyric acid receptor subunit gamma-1](https://go.drugbank.com/polypeptides/Q8N1C3); GABRG2, Gamma-aminobutyric acid receptor subunit gamma-2; GABRG3, Gamma-aminobutyric acid receptor subunit gamma-3; GABRP, [Gamma-aminobutyric acid receptor subunit pi](https://go.drugbank.com/polypeptides/O00591); OXTR, Oxytocin receptor; GABRQ, Gamma-aminobutyric acid receptor subunit theta; HDAC9, **Histone deacetylase 9**.
